# Effectiveness and safety of probiotics in treating knee osteoarthritis: an updated systematic review and meta-analysis of randomized controlled trials

**DOI:** 10.3389/fmed.2026.1799943

**Published:** 2026-06-16

**Authors:** Keye Chen, Yan Sun, Longkang Cui, Yuchen Zhu, Bingbing Zhang, Lianguo Wu

**Affiliations:** Second Affiliated Hospital, Zhejiang Chinese Medical University, Hangzhou, China

**Keywords:** efficacy, gut-joint axis, knee osteoarthritis, meta-analysis, probiotics

## Abstract

Knee osteoarthritis (KOA) is a prevalent degenerative joint disease, and gut microbiota dysbiosis in its pathogenesis is a research hotspot. This study performed a traditional meta-analysis to systematically evaluate probiotics’ efficacy (for KOA symptoms, strain-specific superiority) and safety, using RCT data from Chinese and English databases. Stata 17.0 and RevMan 5.4 analyzed outcomes including patient-reported pain (Visual Analog Scale (VAS), WOMAC pain), joint stiffness, function limitation, serum High-sensitivity C-reactive protein (hs-CRP), and safety. Probiotics showed no significant adverse event differences vs. placebo, with no serious safety issues. Results suggest probiotics have potential for KOA, especially *Saccharomyces boulardii* and *LatiLactobacillus sakei LB-P12*. However, small sample sizes and limited studies weaken evidence; further research is needed.

## 1 Introduction

KOA is one of the most frequent degenerative joint diseases among middle-aged or older people which leads to disabilities by cartilage degeneration. Synovitis, subchondral bone changes, and signs including pain, stiffness and impaired mobility. Existing therapies are unsatisfactory because prolonged use of Non-steroidal anti-inflammatory drugs (NSAIDs) causes damage to many organ systems, glucosamine is of uncertain effectiveness, there exist underdevelopment of Disease-modifying antirheumatic drugs (DMARDs), late stage joint replacement comes with complications (1, 25).

The “gut-joint axis” theory suggests that the imbalance of gut microbiota causes a series of systemic inflammatory responses through the “microbiota-gut-immune” axis—pathogens secrete Lipopolysaccharide (LPS), which passes through the mucosa of the gastrointestinal tract, activates Toll-like receptor 4 (TLR4) signaling, and promotes the release of proinflammatory mediators [Interleukin-6 (IL-6), Tumor necrosis factor-alpha (TNF-α)] in order to accelerate joint inflammation and cartilage degradation (2, 3). KOA patients have gut dysbiosis (higher Bacteroidetes/Firmicutes, lower *Lactobacillus*) and this is related to disease severity (4), suggesting intervention opportunity.

Theoretically, probiotics treat KOA by colonizing the gut and inhibiting pathogens, strengthen the mucosal barrier, regulate immunity, and reduce inflammation. Basic research demonstrates that *Lactobacillus* and *Bifidobacterium* (24) decrease intestinal endotoxin, down-regulate systemic inflammation, and downregulate synovial MMP-13; other strains, such as *LatiLactobacillus sakei LB-P12*, also control Short-chain fatty acid (SCFA) production and Nuclear factor kappa-light-chain-enhancer of activated B cells (NF-κB) through the gut–joint axis (5). Probiotic is safe in use for long-term period on GI disease (6).

Nevertheless, there are still controversies or inconsistencies about the conclusion for KOA with regard to probiotics interventional treatment among existing RCTs and meta-analysis. Particularly, recent meta-analysis draw different conclusion from the same or similar data set. For instance, although Tian et al. (7) find taking pro-biotic could effective to raise WOMAC score and VAS score, Moyseos et al. (8) and Zeng et al. (9) concluded that there was not enough evidence at present to recommend the use of probiotics because of a high risk of bias and methodological limitations. Furthermore, the previous meta-analysis included few trials (only one or two to five), were based on one strain, lacked sufficient heterogeneity tests; thus, it is difficult to draw a conclusion about whether probiotics can be used clinically in patients with KOA.

This research will comprehensively analyze and quantitatively integrate the effectiveness and security of probiotics for KOA based on the conventional meta-analysis method, providing a good reference to use in the clinic or further study.

## 2 Materials and methods

### 2.1 Study design

This narrative and meta-analysis followed the PRISMA statement, using published data without original clinical data collection or participant consent, so no ethics approval was required.

### 2.2 Inclusion and exclusion criteria

The inclusion criteria including: KOA patients (ACR/EULAR criteria, Kellgren-Lawrence ≥ 1, no age/gender/ethnicity limits) receiving single/multi-strain probiotics; control group without probiotics; studies including primary (VAS, WOMAC pain/function) and secondary [serum/synovial Interleukin-1 beta (IL-1β), TNF-α, C-reactive protein (CRP)] outcomes; English/Chinese RCTs, including formally published journal articles and publicly accessible thesis papers approved by academic institutions. The exclusion criteria showed as follows: Non-RCTs, unpublished gray literature (e.g., unfiled internal reports, thesis papers not approved by academic institutions), animal/*in vitro* studies, and intervention groups without probiotics.

### 2.3 Literature search strategy

The literature search strategy was following the PICO principle. P (patients): knee osteoarthritis, knee OA, gonarthrosis; I (Intervention): probiotics, lactobacillus, bifidobacterium; C (Comparator): placebo, usual treatment; O (Outcomes): VAS, WOMAC pain/function, serum/synovial interleukin-1 beta (IL-1β), TNF-α, C-reactive protein (CRP).

The following databases were searched: English (PubMed, Cochrane Library, Web of Science); Chinese (CNKI, Wanfang Data). Period: January 1, 2015–December 30, 2025. The PubMed queries were as follows: ((((knee osteoarthritis) OR (knee OA)) OR (gonarthrosis)) AND (((probiotics) OR (lactobacillus)) OR (bifidobacterium))) AND (((((((VAS) OR (WOMAC function)) OR (WOMAC pain)) OR (serum interleukin-1 beta)) OR (IL-1β)) OR (TNF-α)) OR (C-reactive protein (CRP))).

Supplementary: Included study references and recent systematic reviews/meta-analyses were manually reviewed to identify missed RCTs.

Supplementary explanation: Publicly accessible thesis papers were included in the search process. These papers have been reviewed by the academic committee of the institution, with data collection and analysis processes complying with academic standards, and providing complete research design and original data, which is of great value for supplementing the evidence of this study.

### 2.4 Literature screening and data extraction

Two investigators independently screened titles/abstracts, then full texts, resolving conflicts via discussion or third researcher input. A pre-designed form extracted study characteristics (authors, year, design), participant demographics (sample size), intervention details (strain, dosage, duration), and outcomes (inflammatory markers, Western Ontario and McMaster Universities Osteoarthritis Index (WOMAC), VAS).

### 2.5 Quality assessment of included studies

**Figure 1:**
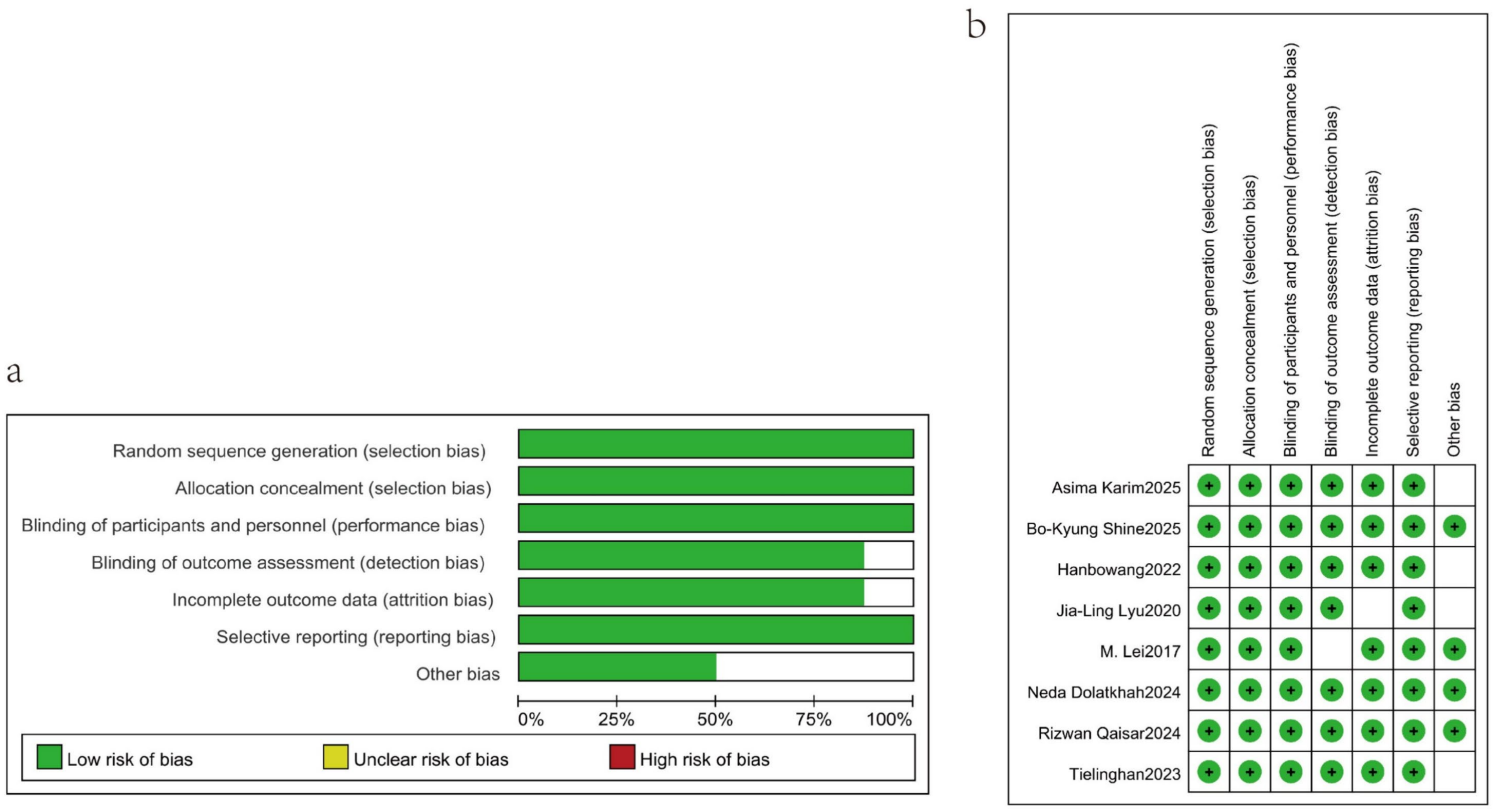
Panel **(a)**: Risk of bias summary; Panel **(b)**: Risk of bias graph.

Most included studies had low bias risk in key domains (random sequence generation, allocation concealment, blinding, selective reporting) per detailed methodological descriptions [e.g., (10, 11)]. A small subset [e.g., (12, 13)] had unclear risk for attrition (incomplete missing data reporting) and other bias (limited confounding variable details). No high bias risk studies were found (Figure 1).

### 2.6 Statistical analysis

RevMan 5.4 and Stata 17.0 were used to analyze data. Heterogeneity was tested via Q test and *I*^2^: fixed-effects was applied if *I*^2^ ≤ 50% and *p* ≥ 0.10, otherwise, the random-effects. For continuous outcomes, weighted mean difference (WMD) was used for outcomes measured by the same tools or standardized mean difference (SMD) when outcomes were measured by different tools. For dichotomous outcomes (adverse events), the Relative risk (RR) was used, all with 95%CI. Sensitivity analysis (sequential study exclusion) and publication bias were assessed by Stata. For core outcomes (VAS pain score, CRP level), publication bias was assessed via Egger’s test and small-study effect regression plots. Funnel plots and trim-and-fill method were assessed when enough studies were included. *p <* 0.05 indicated significant bias.

## 3 Results

### 3.1 Literature search and screening results

**Figure 2:**
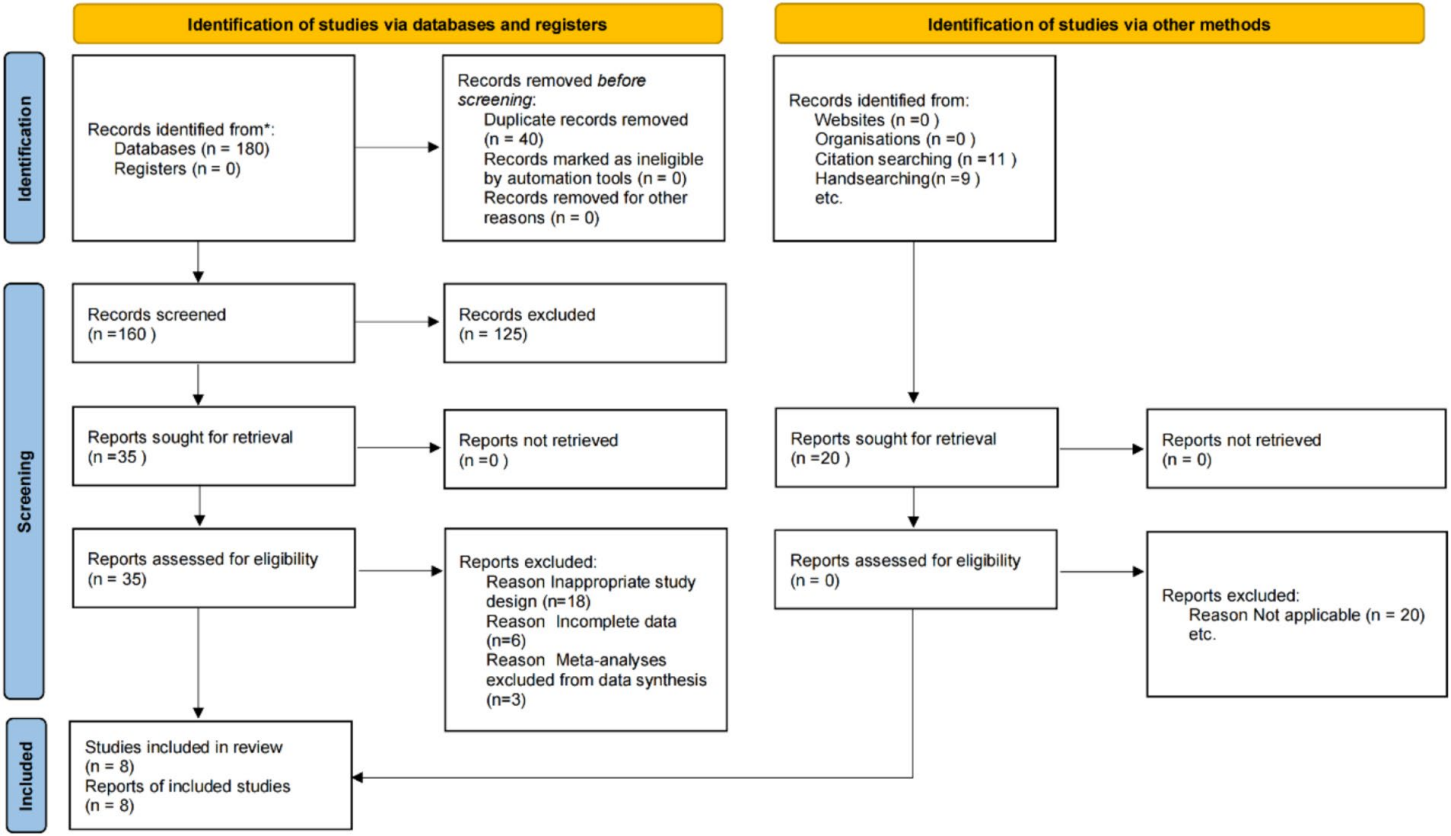
Literature search and screening results. Initial search and supplementary search yielded 180 + 20 = 200 records. After excluding 40 duplicate records, 160 records were screened; 125 records were excluded due to irrelevant topics/designs, unpublished gray literature, etc., leaving 35 for full-text evaluation; 27 were excluded due to inappropriate study design, incomplete data, or being meta-analyses excluded from data synthesis, and finally 8 studies were included.

Records identified through the search processes (*n =* 180) and other sources (*n =* 20: manual = 9, reference tracing = 11). After removal of duplicates (*n =* 40), 160 studies were included in the screening process. After 125 irrelevant studies excluded from study, 35 studies were left for full-text assessment. Of theses, 27 were excluded because of inappropriate study design, incomplete data, or because they were meta-analyses excluded from data synthesis. Finally, eight studies met the criteria (Figure 2).

### 3.2 Basic characteristics of included studies

Eight RCTs (10–17) whose basic demographic data were similar and be included in the analysis of this review. The intervention group used one kind of strain (*Lactobacillus casei Shirota*), multi-strains (Vivomix 8), or a combination of probiotics and glucosamine/physical therapy. The control using placebo with adjunct intervention. Duration of the intervention was 3–6 months with the main results including hs-CRP, WOMAC, VAS (Table 1).

**Table 1:** Basic characteristics of included studies.

Author (year)	Sample size (N)		Intervention		Period	Outcomes
	Study	Control	Study	Control		
Lei et al. (12)	215	218	*Lactobacillus casei Shirota*	Placebo	6 months	High-sensitivity C-reactive protein (hsCRP); WOMAC; Visual Analog Scale (VAS)
Lyu et al. (13)	37	30	TCI633	Placebo	12 weeks	hs-CRP; WOMAC
Wang (14)	37	28	*Bifidobacterium lactis* + calcium+ chondroitin	Placebo + calcium+ chondroitin	4 months	WOMAC; Inflammatory factors; Bone density
Han (15)	32	34	*Bifidobacterium* + glucosamine	Placebo + glucosamine	3 months	WOMAC; VAS; Inflammatory factors; Body mass index (BMI)
Dolatkhah et al. (16)	32	31	*S. boulardii* + physical therapy	Placebo + physical therapy	12 weeks	hs-CRP; WOMAC; VAS; Inflammatory factors
Shine et al. (11)	45	47	*LatiLactobacillus sakei LB-P12*	Placebo (carboxymethyl cellulose)	12 weeks	WOMAC; VAS; Inflammatory factors; Joint space width
Karim et al. (17)^b^	65	58	Vivomix (8strains)^a^	Placebo (fruit powder)	12 weeks	WOMAC; VAS; Inflammatory factors; Bone density
Karim et al. (10)	46	49	Vivomix (8strains)^a^	Placebo	16 weeks	VAS; Physical function; Inflammatory factors

a Vivomix (8 strains): *Streptococcus thermophilus* DSM 24731, *Bifidobacterium longum* DSM 24736, *B. breve* DSM 24732/24737, *Lactobacillus* spp. DSM 24735/24730/24733, *L. delbrueckii* subsp. *bulgaricus* DSM 24734.

b The study labeled as “Rizwan Qaisar 2024” in the forest plots corresponds to the same clinical trial as Karim et al. (17), with identical patient cohorts and outcome data. The discrepancy in first authorship is due to different author orderings in the publication and dataset presentation.

### 3.3 Meta-analysis, publication bias and small-study effect analysis

#### 3.3.1 Pain score improvement

##### 3.3.1.1 VAS pain

**Figure 3:**
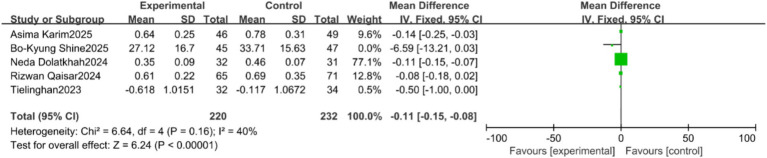
VAS pain scores.

Five studies showed that probiotics significantly reduced VAS scores vs. controls (MD = −0.11, 95%CI: −0.15 to −0.08, *Z* = 6.24, *p <* 0.00001; Figure 3).

##### 3.3.1.2 Publication bias and small-study effect (VAS pain)

**Figure 4:**
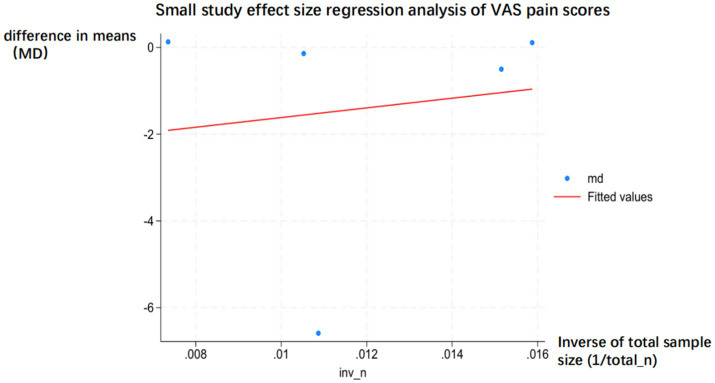
Small-study effect regression analysis of VAS pain scores. Each scatter point represents one RCT, and the red line is the regression fit line. The regression analysis showed *p =* 0.828, indicating no significant small study effect.

Egger’s test for VAS pain (Table 2) suggested no obvious publication bias (*p =* 0.054 > 0.05). Small-study effect regression analysis (Figure 4) indicated no obvious small-study bias (*p =* 0.828 > 0.05). Combined with the results of sensitivity analysis, the robustness of the results of this outcome indicator was initially supported, but it still needed to be interpreted with caution.

**Table 2:** Results of Egger’s regression test for publication bias.

Metric	Coefficient	Standard error	t price	P price	95% confidence interval
Slope	0.1478	0.0481	3.07	0.054	−0.0053 ~ 0.3008
Bias	−2.3347	1.1536	−2.02	0.136	−6.0058 ~ 1.3365

Test of H_0_: no small-study effects, *p* = 0.136.

Supplementary explanation: Due to the number of RCTs included for core outcome indicators (VAS pain score, CRP level) being only 5 and 4 studies respectively, and the applicability of funnel plots and the trim-and-fill method is limited, so these methods were not used for publication bias assessment. The results of the publication bias analysis in this study are limited by the sample size and have certain limitations; future studies need to include more high-quality RCTs for further verification.

##### 3.3.1.3 WOMAC pain

**Figure 5:**
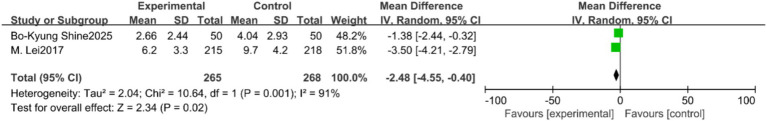
WOMAC pain scores.

Two studies (268 participants) showed probiotics lowered WOMAC pain scores (MD = −2.48, 95%CI: −4.55 to −0.40, *Z* = 2.34, *p =* 0.02) with high heterogeneity (*I*^2^ = 91%, *p =* 0.001; random-effects model; Figure 5).

#### 3.3.2 Joint function score improvement

##### 3.3.2.1 WOMAC total

**Figure 6:**
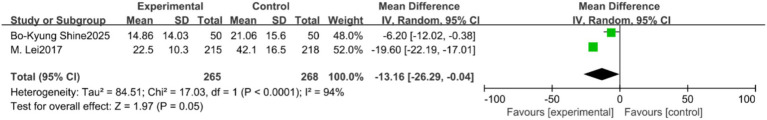
WOMAC total scores.

Two studies (268 participants) showed slight but significant WOMAC total score reduction with probiotics (random-effects model; Figure 6).

##### 3.3.2.2 WOMAC stiffness

**Figure 7:**
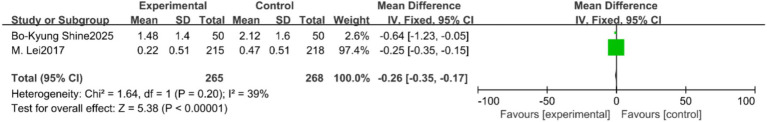
WOMAC stiffness score.

Two studies (268 participants) showed probiotics reduced stiffness scores (MD = −0.26, 95%CI: −0.35 to −0.17, *Z* = 5.38, *p <* 0.00001; Figure 7).

##### 3.3.2.3 WOMAC physical function

**Figure 8:**
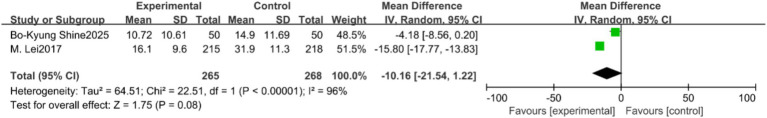
WOMAC physical function score.

Two studies (268 participants) showed no significant improvement (MD = −10.16, 95%CI: −21.54 to 1.22, *Z* = 1.75, *p =* 0.08) with high heterogeneity (*I*^2^ = 96%, *p <* 0.00001; random-effects model; Figure 8).

#### 3.3.3 Inflammatory cytokine changes

**Figure 9:**
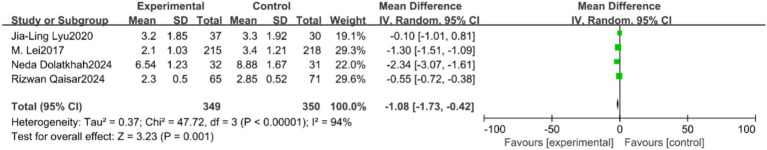
Changes in inflammatory cytokine levels.

Four studies (350 patients) showed probiotics reduced serum CRP (MD = −1.08, 95%CI: −1.73 to −0.42, *Z* = 3.23, *p =* 0.001) with high heterogeneity (*I*^2^ = 94%, *p <* 0.00001; random-effects model; Figure 9).

##### 3.3.3.1 Publication bias and small-study effect (CRP)

**Figure 10:**
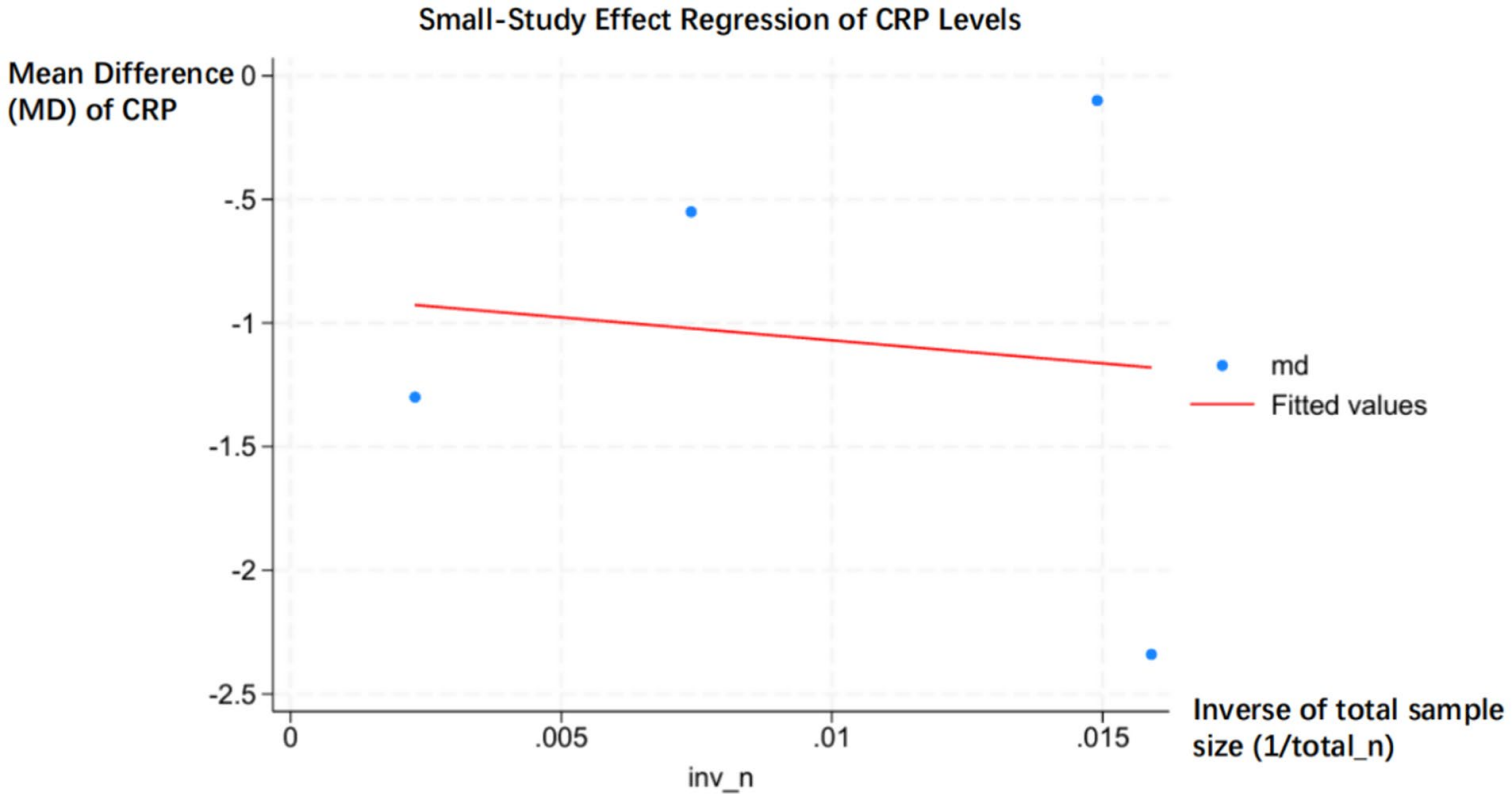
Small-study effect regression of CRP. Each scatter point represents one RCT, and the red line is the regression fitted line. The regression analysis showed *p =* 0.680, indicating no significant small—study effect.

Egger’s test for CRP (Table 3) showing no obvious publication bias (*p =* 0.680 > 0.05). Small-study effect regression analysis (Figure 10) indicating no small-study bias (*p =* 0.680 > 0.05). The robustness of the results needs to be further confirmed with more research data.

**Table 3:** Results of Egger’s regression test for publication bias.

Metric	Coefficient	Standard error	t price	P price	95% confidence interval
Slope	−0.6503	0.5665	−1.15	0.37	−3.0878 ~ 1.7872
Bias	−2.0814	4.3528	−0.48	0.68	−20.8104 ~ 16.6475

Test of H₀: no small-study effects, *p* = 0.680.

#### 3.3.4 Adverse event analysis

**Figure 11:**
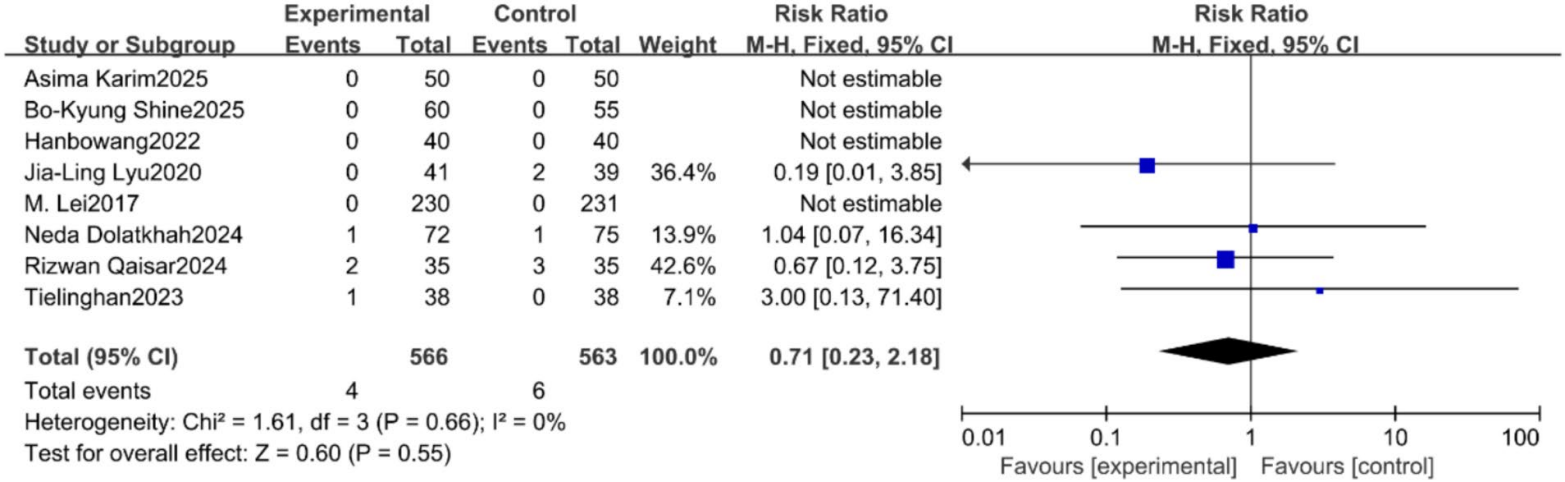
The forest plot of adverse event analysis.

Eight studies (1,129 patients) showed no significant difference in adverse event incidence between probiotics and placebo (RR = 0.71, 95%CI: 0.23–2.18, *Z* = 0.60, *p =* 0.55). The result was shown in Figure 11.

#### 3.3.5 Strain-specific efficacy

Subgroup analysis of 8 RCTs (strains: *Lactobacillus brevis* LB-P12, *Streptococcus thermophilus* TCI633, Vivomix, *Bifidobacterium infantis* BX-01, *Lactobacillus casei Shirota*, *Saccharomyces boulardii*, *Bifidobacterium lactis* Probio-M8) showed that.

**Figure 12:**
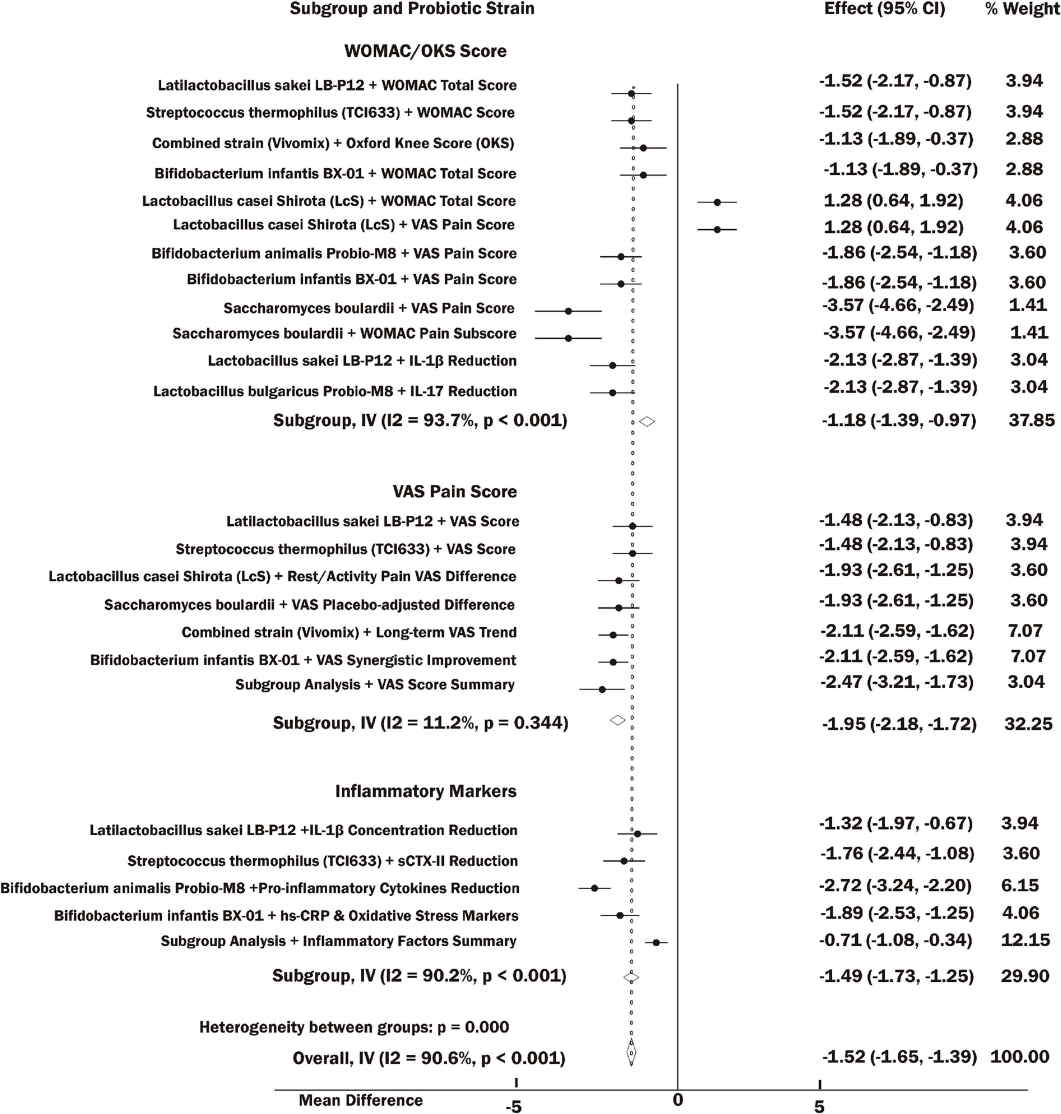
Comparative efficacy among different strains.

*Saccharomyces boulardii* had the strongest analgesic effect (WOMAC pain MD = -3.57, 95%CI: −4.66 to −2.49) (16). *Bifidobacterium infantis* BX-01 (VAS MD = −2.11, hs-CRP MD = −1.89) and *LatiLactobacillus sakei LB-P12* (WOMAC total MD = −1.52, VAS MD = −1.48, IL-1β MD = −1.32) excelled in symptom relief + inflammation regulation (11). Moderate heterogeneity (e.g., WOMAC/OKS *I*^2^ = 93.7%) was linked to strain, dosage, and duration (Figure 12).

### 3.4 Sensitivity analysis and heterogeneity of WOMAC outcomes

**Figure 13:**
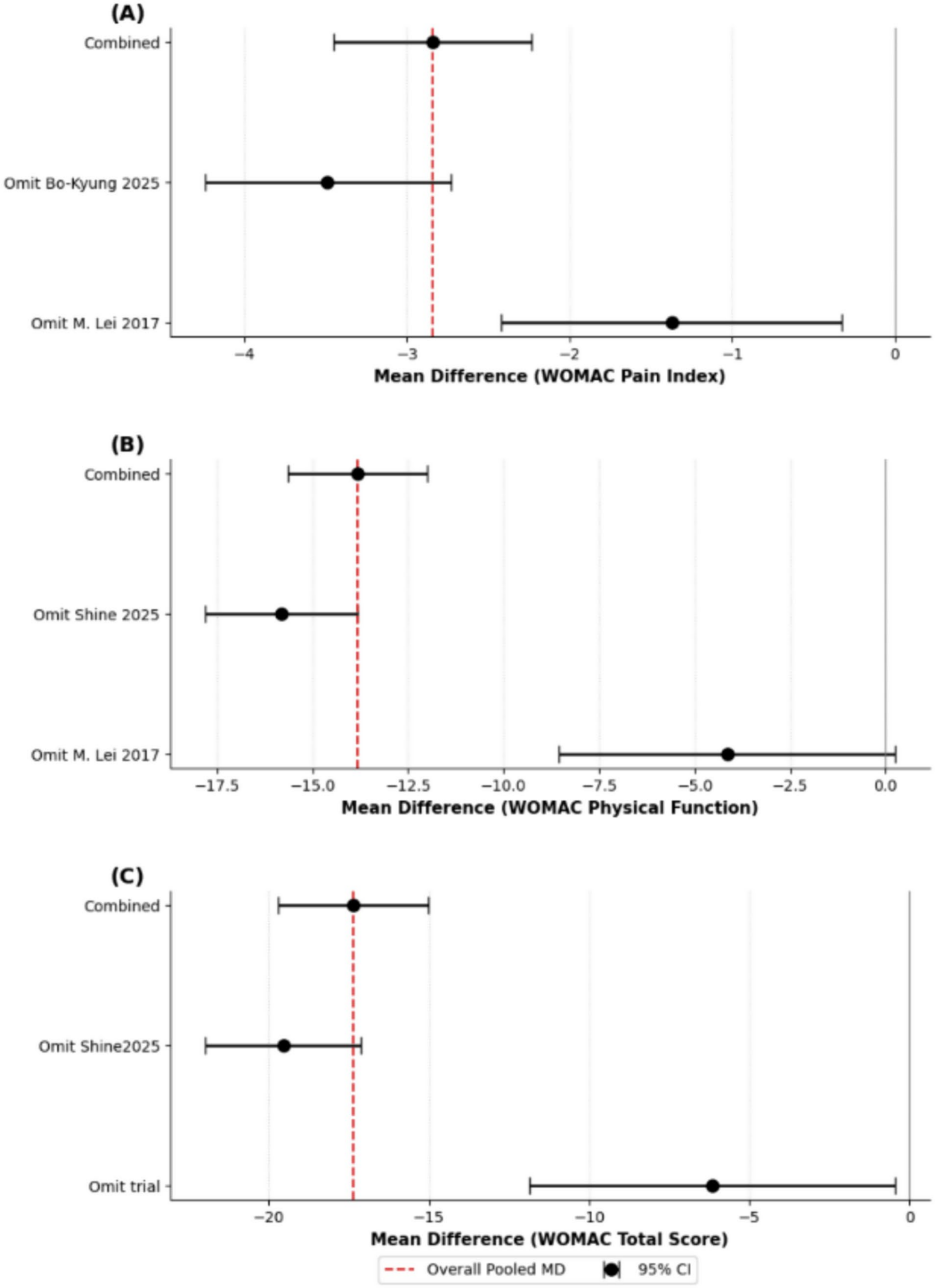
Leave-one-out analysis (**A**: WOMAC Pain Index; **B**: WOMAC Physical Function; **C**: WOMAC Total Score).

Leave-one-out sensitivity was shown in Figure 13 (*N =* 2). Result showed a very high sensitivity after removing Lei et al. (12) causing effect toward null, and after removing Shine et al. (11) the effect changed toward the opposite. Such dramatic change suggested that there was large amount of clinical heterogeneity instead of chance noise. In order to figure out what caused such large heterogeneity, we compared the main features between those two critical papers (Table 4).

**Table 4:** Sources of clinical and methodological heterogeneity.

Domain	Indicator	Lei et al. (12)	Shine et al. (11)	Impact on heterogeneity
Baseline severity	WOMAC scores (*total, pain, function*)	High	Low	Marked baseline disparity across all dimensions induced a systemic ceiling effect, uniformly limiting absolute improvement in the Shine cohort
Disease stage	KL grade	Grade 2–3	Grade 1–2	Earlier structural stage correlates with attenuated therapeutic responsiveness in both pain and function domains
Inflammatory mechanism	Primary biomarker	hs-CRP ↓ (systemic)	IL-1β ↓ (local)	Divergent mechanistic pathways (systemic vs. local) create non-comparable biological endpoints
Intervention duration	Timeframe	24 weeks	12 weeks	Shorter duration likely precludes cumulative functional benefits, contributing to variance in long-term outcomes

For the main features compared in Table 4, we found the following four reasons causing heterogeneity: (1) Baseline severity: more severe state of illness existing in study of Lei et al. causing “ceiling effect” in Shine et al.; (2) Stage of disease: different fibrosis (K-L grade 2 to 3) or different levels of inflammation; (3) Type of marker: inflammatory factors were hsCRP or cytokine (IL-1b); (4) Treatment duration: 24 or 12 weeks. All these factors may account for a part of the high between-study variability.

### 3.5 Subgroup and meta-regression analysis

There were only two studies included in the comparison of WOMAC scores (pain, function, total) with considerable heterogeneities, and the subgroup analyses were unable to perform. For CRP, we did the meta-regression analysis, and the result showed that the mean ages were the source of the heterogeneity (*Z* = 1.99, *p* = 0.046).

**Figure 14:**
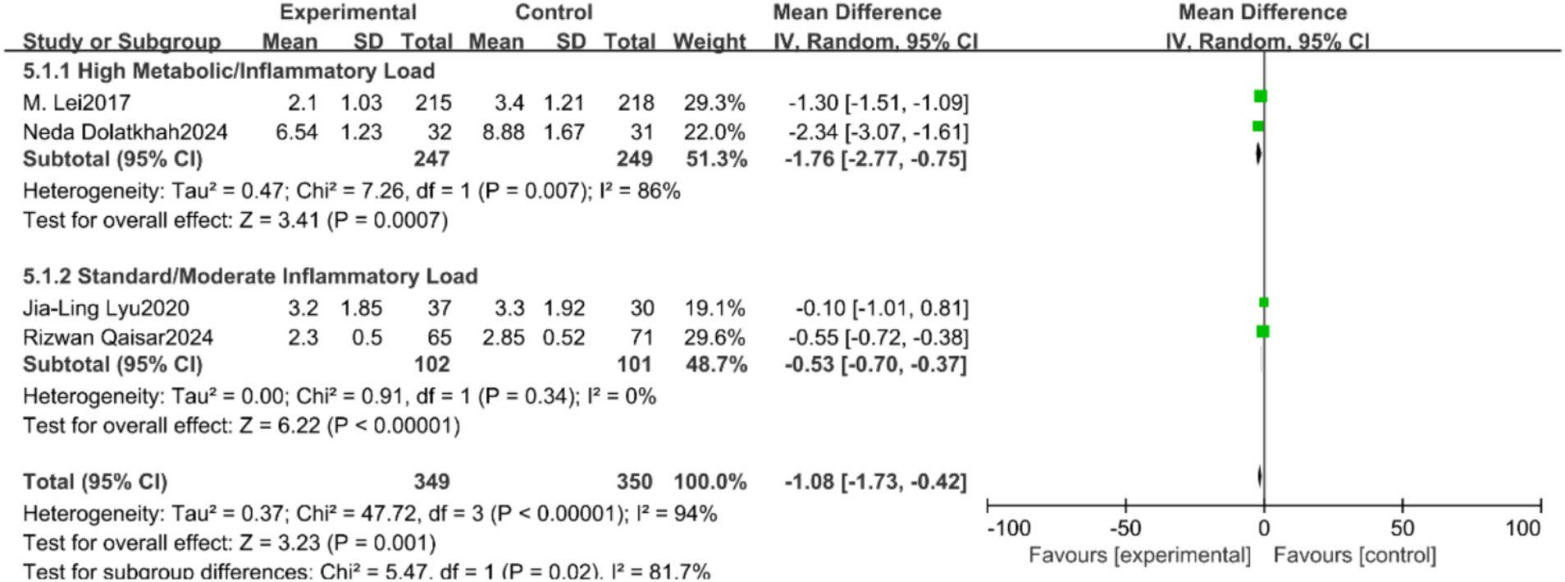
Sources of heterogeneity in CRP outcomes: Subgroup analysis by baseline metabolic load.

In subgroup analysis of CRP, the treatment effect was different to some extent regarding the initial metabolism burden (Figure 14). In high burden group, there were more obvious reductions of CRP (MD = −1.76; 95% CI: −2.77a to −0.75d; *I*^2^ = 86%) while in the normal-load subgroup, we found a moderate homogeneous effect (MD = −0.53; 95% CI: −0.70 to −0.37; *I*^2^ = 0%). The heterogeneity between subgroups (*p =* 0.02) suggested pre-existing inflammatory status accounting for most variability.

To understand the heterogeneity (*I*^2^ = 86%) within the high load group, we found three sources of evidence (Table 5), (1) Taxonomy: different probiotics in study group (bacteria *L. casei* vs. fungi *S. boulardii*); (2) Time course: different therapy time (24 weeks vs. 12 weeks) and (3) Initial BMI: different adipokines-induced inflammatory responses between the restricted-weight group vs. non-restricted weight group. This could explain some of the inconsistency.

**Table 5:** Clinical heterogeneity analysis in subgroup with high metabolic/inflammatory load.

Parameter	Lei et al. (12)	Dolatkhah et al. (16)	Proposed mechanism for CRP heterogeneity
Probiotic strain	*Lactobacillus casei Shirota*	*Saccharomyces boulardii*	Distinct immunomodulatory pathways between bacterial and fungal kingdoms
Intervention duration	24 weeks (6 months)	12 weeks (3 months)	Longer duration may enhance cumulative anti-inflammatory effects
Baseline BMI status	Not restricted	Overweight/obese	Adipokine-mediated chronic inflammation may drive divergent CRP responses

CRP, C-reactive protein.

## 4 Discussion

Probiotics decreased VAS/WOMAC pain/stiffness score and modulated inflammation markers (CRP, IL-6, TNF-α) similar to the pain/function results (3, 18). This indicates that probiotics block the systemic inflammation in order to reduce the joint inflammation and cartilage damage, to achieve “anti-inflammation→analgesia→function improvement.”

Mechanistically, this is in line with a “gut-joint axis,” which describes how probiotics may provide systemic immunomodulation and metabolic reprogramming. The mechanisms seem to involve at least two main pathways: First, immunomodulation by T-cell polarization: some strains such as *Lactobacillus casei Shirota* induce the differentiation of regulatory T cells (Tregs), which contributes to the establishment of an antiinflammatory environment (6). *S. boulardii* improve intestinal permeability in order to avoid translocation of inflammatory pathogens triggering macrophages activation (19). Secondly, metabolic signal through microbiota derived metabolite: Interestingly, *LatiLactobacillus sakei LB-P12* regulates short chain fatty acids (SCFAs) production and SCFAs down regulate NF-κB pathway by blocking histone deacetylases (HDACs), thus inhibiting catabolic enzyme (e.g., MMP-13), proinflammatory cytokine (e.g., IL-1ß, IL-6) production in the joint thus slowing down the rate of cartilage degradation.

Probiotics did not significantly impact WOMAC physical function (MD = −10.16, *p =* 0.08) likely due to the fact that functional recovery may require structural recovery (e.g., cartilage) and muscle strength improvement—probiotics affect inflammation/symptoms—not structure; short duration of interventions (8–24 weeks)—also might contribute indicating a requirement of longer follow-ups.

### 4.1 Therapeutic potential and underlying mechanisms of probiotics in KOA

Vasomotor symptoms were also decreased by probiotics as was the inflammation reflected in CRP, IL-6, and TNF-α; these findings paralleled those seen for pain/function (3, 18). Thus, it appears that the action of probiotics consists on inhibiting systemic inflammation aiming at reducing joint inflammation and cartilage damage, achieve “anti-inflammatory → analgesia → function improvement (26, 27).” The effects of probiotics were not statistically significant in WOMAC physical function (MD = −10.16, *p =* 0.08) possibly because there was high clinical heterogeneity. Different baseline severity and different structural stage among trials would dilute the pooled estimate. Probiotics mainly targeting inflammation instead of structural repair mayand the duration of interventions (8–24 weeks), were low, detectable functional improvements were limited.

Clinically, simultaneous amelioration on both pain and stiffness with reduction of CRP indicated that the application of probiotics may be able to modulate multiple aspects simultaneously in KOA. Although the overall magnitude of effects was relatively small, the larger effect of CRP lowering seen with the “higher metabolic / inflammatory burden” group (MD = −1.76), indicates that there may be differential responses based on particular clinical phenotype, and also preliminary safety data (RR = 0.71) suggest that the administration of probiotics is well tolerated as add on treatment. These results show a possible use for their application to reduce general inflammatory response, the ultimate clinical benefit needs to be validated by high quality trials.

### 4.2 Clinical significance and practical implications

The reported decreases of WOMAC total score (MD = −13.16; 95% CI: −26.29 to −0.04), and stiffness (MD = −1.13; 95% CI: −1.43 to −0.83) seem plausible given the Minimal clinically important difference (MCID) range (−7.0 to 14.6 points on total score and −0.5 to −1.6 point on stiffness) (20). This implies that the probiotic treatment might provide an effective degree of symptom alleviation, which could be experienced by KOA patients. Although this improvement of physical function (MD = −10.16) was not statistically significant (*p >* 0.05), it is still inside the range of MCID, which may indicate a potential clinical tendency. In addition, despite the relatively small numeric change of VAS pain (MD = −0.11 cm), the large reduction in hsCRP (SMD = −1.14) indicates that the mechanism by which probiotics work could be via systemic anti-inflammation and not directly as a strong analgesic agent. Therefore, as a clinical-relevant adjuvant treatment, it is possible that probiotics could be valuable in the multi-dimensional and long-term management of KOA.

### 4.3 Strain-specificity

Subgroup analysis identified differential strain effects: *Lactobacillus casei Shirota* (VAS MD = −2.65, WOMAC total MD = −2.70, low heterogeneity) was optimal (9, 12); *Saccharomyces boulardii* focused on analgesia (16).

*Bifidobacterium infantis* BX-01 and *LatiLactobacillus sakei LB-P12* combined symptom relief and anti-inflammation (11). These differences may relate to metabolites, gut colonization, and immunomodulation (e.g., *Lactobacillus casei Shirota* regulates Treg cells; *Saccharomyces boulardii* protects intestinal mucosa) (6, 19).

### 4.4 Sensitivity analysis and reliability

Sensitivity analysis (leave-one-out) shows that VAS and WOMAC estimations are stable without any single study having a strong impact on the results; heterogeneity mainly comes from different intervention protocols (strain, dose, and duration); trials with consistent strain-specific protocols [e.g., (9)] exhibit lower variability. Furthermore, the favorable safety profile (AE incidence: probiotics 0.71% vs. placebo 1.07%, *p =* 0.55) suggests good tolerance for probiotic usage. Although promising regarding the alleviation of symptoms more research is necessary to obtain consistency and reproducibility of large scale RCTs.

### 4.5 Strengths, limitations, and future directions

#### 4.5.1 Strengths

Compared with other recent reviews (7–9), this review may give broader perspective because it includes more studies in term of nRCTs (*n =* 8), which can increase power to detect KOA specific responses. One of our strengths will be an effort to bring together the areas of pooled efficacy with those of clinical use in a finer grain, strain-specific manner (e.g., S*. boulardii* vs. LB-P12). In addition, through defining “high metabolic/inflammatory load” group as a high-response group (CRP SMD = −1.76), we provided some initial information about individualized clinical decisions which are not widely discussed in previous meta-analyses (Table 6).

**Table 6:** Comparison of methodological characteristics and key findings between the present study and previous meta-analyses.

Methodological dimension	Zeng et al. (9)	Moyseos et al. (8)	Tian et al. (7)	Present study
Scope and focus	Umbrella (8 arthritis types)	OA/RA specific	KOA specific	KOA specific
No. of KOA RCTs	1	3	5	**8**
Strain-specific analysis	None (single strain)	Limited (LcS focus)	None (pooled effect)	**Yes (SB vs. LB-P12)**
Heterogeneity management	Superficial	Identified bias	Sensitivity only	**In-depth (subgroups)**
Key conclusion	Insufficient evidence	LcS effective	Overall efficacy	**Strain-specific + mechanism**

SB, *Saccharomyces boulardii*; LB-P12, *LatiLactobacillus sakei* LB-P12. Bold text indicates the unique strengths and novel contributions of the current study relative to prior research.

#### 4.5.2 Limitations

Limited sample size of some outcome (e.g., BMI/BMD), few study about specific strain (e.g., *Streptococcus thermophilus* TCI633) (13), and no long-term KOA progressions. In addition, only eight RCTs were included (<10), therefore the publication bias was tested by Egger’s test and small-study effect regression plot, with trim-and-fill analysis (≥10 studies) unavailable. Another limitation, the evaluation of publication bias in this article also exists some shortcomings. For core outcomes indicator, there are less than 10 RCTs included, leading to poor statistical power of methods like Egger’s test that are unable to fully exclude possible influence from publication bias, and one has to be careful in interpreting the corresponding result. Therefore, it is important to note that any beneficial effects seen in symptoms cannot be taken as a true demonstration of efficacy.

#### 4.5.3 Future research should

Conduct large-sample, long-term RCTs on priority strains (*Lactobacillus casei Shirota*, *Bifidobacterium infantis* BX-01) to assess long-term joint protection (11, 12, 28).

Use gut microbiome sequencing/metabolomics to clarify strain-specific molecular mechanisms (21, 22).

Explore personalized interventions (age, KL grade, baseline microbiota) and build clinical decision models for strain selection (9, 23).

## 5 Conclusion

Probiotic relieves the pain of KOA (VAS, WOMAC pain), improves joint function, reduces serum inflammation, and has a safety similar to placebo, with *Saccharomyces boulardii* (16) and *LatiLactobacillus sakei LB-P12* (11) showing promising results. However, smaller study size and fewer trials dilute strength; further they are clinically relevant only to a lesser extent due to smaller magnitudes of effects. Thus, there is a need for large-sample, high quality RCT’s that can help confirm both efficacy and strain-specificity.

## Data Availability

The original contributions presented in the study are included in the article/supplementary material, further inquiries can be directed to the corresponding author.

## References

[1] Geng R Li J Yu C Zhang C Chen F Chen J . Knee osteoarthritis: current status and research progress in treatment (review). Exp Ther Med. (2023) 26:481. doi: 10.3892/etm.2023.12180, 37745043 PMC10515111

[2] Deng HL Tang ZM. Cartilage-intestine-microbiome axis: Progress in a novel model for the treatment of osteoarthritis. Chin J Bone Joint Injury. (2024) 39:832–6. (In Chinese)

[3] Amin U Jiang R Raza SM Fan M Liang L Feng N . Gut-joint axis: Oral probiotic ameliorates osteoarthritis. J Tradit Complement Med. (2024) 14:26–39. doi: 10.1016/j.jtcme.2023.06.002, 38223812 PMC10785157

[4] Wang X Wu Y Liu Y Chen F Chen S Zhang F . Altered gut microbiome profile in patients with knee osteoarthritis. Front Microbiol. (2023) 14:1153424. doi: 10.3389/fmicb.2023.1153424, 37250055 PMC10213253

[5] Song M Kim WJ Shim J Song K. *LatiLactobacillus sakei LB-P12* ameliorates osteoarthritis by reducing cartilage degradation and inflammation via regulation of NF-kappaB/HIF-2alpha pathway. J Microbiol Biotechnol. (2025) 35:e2504013. doi: 10.4014/jmb.2504.04013, 40329628 PMC12089955

[6] Ferrillo M Giudice A Migliario M Reno F Lippi L Calafiore D . Oral-gut microbiota, periodontal diseases, and arthritis: literature overview on the role of probiotics. Int J Mol Sci. (2023) 24:4626. doi: 10.3390/ijms24054626, 36902056 PMC10003001

[7] Tian M Zhu Y Lu S Qin Y Li X Wang T . Clinical efficacy of probiotic supplementation in the treatment of knee osteoarthritis: a meta-analysis. Front Microbiol. (2025) 16:1526690. doi: 10.3389/fmicb.2025.1526690, 40276226 PMC12020436

[8] Moyseos M Michael J Ferreira N Sophocleous A. The effect of probiotics on the management of pain and inflammation in osteoarthritis: a systematic review and meta-analysis of clinical studies. Nutrients. (2024) 16:2243. doi: 10.3390/nu16142243, 39064686 PMC11279588

[9] Zeng L Deng Y He Q Yang K Li J Xiang W . Safety and efficacy of probiotic supplementation in 8 types of inflammatory arthritis: a systematic review and meta-analysis of 34 randomized controlled trials. Front Immunol. (2022) 13:961325. doi: 10.3389/fimmu.2022.961325, 36217542 PMC9547048

[10] Karim A Khan HA Ahmad F Qaisar R. Probiotics improve functional performance in patients with osteoarthritis: a randomized placebo-controlled clinical trial. Eur J Nutr. (2025) 64:290. doi: 10.1007/s00394-025-03805-8, 41065805

[11] Shine BK Li Q Song M Song K Shim J Han SH. Efficacy and safety of *LatiLactobacillus sakei LB-P12* in patients with knee osteoarthritis: an exploratory randomized, double-blind, placebo-controlled clinical trial. Sci Rep. (2025) 15:25980. doi: 10.1038/s41598-025-11250-0, 40676109 PMC12271348

[12] Lei M Guo C Wang D Zhang C Hua L. The effect of probiotic *Lactobacillus casei Shirota* on knee osteoarthritis: a randomised double-blind, placebo-controlled clinical trial. Benef Microbes. (2017) 8:697–703. doi: 10.3920/BM2016.0207, 28726510

[13] Lyu JL Wang TM Chen YH Chang ST Wu MS Lin YH . Oral intake of Streptococcus thermophil us improves knee osteoarthritis degeneration: a randomized, double-blind, placebo-controlled clinical study. Heliyon. (2020) 6:e03757. doi: 10.1016/j.heliyon.2020.e03757, 32368640 PMC7184258

[14] Wang HB. Clinical Study on Probiotics Adjuvant Treatment of Postmenopausal Women with Osteoporosis and Knee Osteoarthritis. Hohhot: Inner Mongolia Medical University (2022) (Master's Thesis, In Chinese).

[15] Han TL. The Clinical Study of Probiotics in Adjunctive Therapy on Knee Osteoarthritis. Hohhot: Inner Mongolia Medical University (2023) (Master's Thesis, In Chinese).

[16] Dolatkhah N Jafari A Eslamian F Toopchizadeh V Saleh P Hashemian M. *Saccharomyces boulardii* improves clinical and paraclinical indices in overweight/obese knee osteoarthritis patients: a randomized triple-blind placebo-controlled trial. Eur J Nutr. (2024) 63:2291–305. doi: 10.1007/s00394-024-03428-5, 38761281

[17] Karim A Khan HA Iqbal MS Ahmad F Qaisar R. Probiotics' supplementation alleviates disease severity and improves postural balance by repairing intestinal leak in patients suffering from osteoarthritis: a double-blinded clinical trial. Br J Nutr. (2024) 132:1602–10. doi: 10.1017/S0007114524002824, 39523206

[18] Corriero A Giglio M Soloperto R Inchingolo F Varrassi G Puntillo F. Microbial symphony: exploring the role of the gut in osteoarthritis-related pain. A narrative review. Pain Ther. (2024) 13:409–33. doi: 10.1007/s40122-024-00602-9, 38678155 PMC11111653

[19] Rahman SO Bariguian F Mobasheri A. The potential role of probiotics in the Management of Osteoarthritis Pain: current status and future prospects. Curr Rheumatol Rep. (2023) 25:307–26. doi: 10.1007/s11926-023-01108-7, 37656392 PMC10754743

[20] Silva MDC Perriman DM Fearon AM Couldrick JM Scarvell JM. Minimal important change and difference for knee osteoarthritis outcome measurement tools after non-surgical interventions: a systematic review. BMJ Open. (2023) 13:e063026. doi: 10.1136/bmjopen-2022-063026, 37202126 PMC10201231

[21] Di J Xi Y Wu Y Di Y Xing X Zhang Z . Gut microbiota metabolic pathways: key players in knee osteoarthritis development. Exp Gerontol. (2024) 196:112566. doi: 10.1016/j.exger.2024.112566, 39226947

[22] Zhu D Wang X Xi Z Chen K Feng Y Zi C . Diet influences knee osteoarthritis osteophyte formation via gut microbiota and serum metabolites. iScience. (2024) 27:110111. doi: 10.1016/j.isci.2024.110111, 38957790 PMC11217616

[23] Wang Z Zhao C Wang Z Li M Zhang L Diao J . Elucidating causal relationships among gut microbiota, human blood metabolites, and knee osteoarthritis: evidence from a two-stage Mendelian randomization analysis. Rejuvenation Res. (2025) 28:239–47. doi: 10.1089/rej.2024.0079, 40193247

[24] Wang K Wang H Zhao Z Shen X Zhao J Zhang H. *Bifidobacterium animalis* subsp. Lactis Probio-M8 enhances chondroitin efficacy for knee osteoarthritis in postmenopausal women via the gut-joint axis. mSystems. (2025) 10:e0086225. doi: 10.1128/msystems.00862-25 41313018 PMC12710372

[25] Korotkyi O Vovk A Kuryk O Dvorshchenko K Falalyeyeva T Ostapchenko L . Co-administration of live probiotics with CHONDROPROTECTOR in management of experimental knee osteoarthritis. Georgian Med News. (2018) 279:191–6. 30035745

[26] Mladenova I. The potential involvement of human microbiota in osteoarthritis: encouraging data useful for future studies. Minerva Med. (2019) 110:399–400. doi: 10.23736/S0026-4806.19.06147-0, 31124640

[27] Jhun J Min HK Na HS Kwon JY Ryu J Cho KH . Combinatmarion treatment with *Lactobacillus acidophilus* LA-1, vitamin B, and curcumin ameliorates the progression of osteoarthritis by inhibiting the pro-inflammatory mediators. Immunol Lett. (2020) 228:112–21. doi: 10.1016/j.imlet.2020.10.008, 33137380

[28] Taye I Bradbury J Grace S Avila C. Probiotics for pain of osteoarthritis; an N-of-1 trial of individual effects. Complement Ther Med. (2020) 54:102548. doi: 10.1016/j.ctim.2020.102548, 33183666

